# The role of E-cadherin and β-catenin in laryngeal cancer

**DOI:** 10.18632/oncotarget.25680

**Published:** 2018-07-10

**Authors:** Carlos Eduardo Nardi, Rogério Aparecido Dedivitis, Ricardo Camillo de Almeida, Leandro Luongo de Matos, Claudio Roberto Cernea

**Affiliations:** ^1^ Department of Head and Neck Surgery, Hospital das Clínicas, São Paulo School of Medicine, São Paulo, Brazil; ^2^ Pathologist, Clínica Diagnos, Santos, Brazil; ^3^ Department of Head and Neck Surgery, São Paulo School of Medicine, Assistant, Instituto do Câncer do Estado de São Paulo, ICESP (São Paulo State Cancer Institute), São Paulo, Brazil; ^4^ Department of Head and Neck Surgery, São Paulo School of Medicine, University of São Paulo, São Paulo, Brazil

**Keywords:** cadherins, beta-catenin, laryngectomy, laryngeal neoplasms, carcinoma

## Abstract

Epithelial cadherins with catenins form the E-cadherin-catenin complex that acts on cell-to-cell adhesion. The loss of these complex lead to the reduction or absence of epithelial cadherin expression in the cell membrane, cytoplasmic accumulation of β-catenin and its translocation to the nucleus, contributing to carcinogenic events. The objective of this study was to evaluate the expression of epithelial cadherin and β-catenin in patients with laryngeal tumor. A retrospective study of 52 patients with glottic or supraglottic squamous cell carcinoma was conducted and evaluated according to the tumor site, histological differentiation, TNM stage, survival analysis and compared with the immunohistochemical expression of epithelial cadherin and β-catenin. We observed statistically significant association between the epithelial cadherin expression reduction and supraglottic localization of the lesion, the presence of cervical metastasis, poorly differentiated tumors and locally advanced tumors when in glottic topography. Related to the expression of β-catenin, statistical significance was also found to the presence of cervical metastasis and tumor of low differentiation with the decreased expression of this marker. Regarding survival analysis, the low expression of β-catenin is related to worse overall survival and the reduction of expression of both markers to worse disease-free survival. We concluded that the reduction in expression of the markers studied leads to a prognostic impact as they are related to tumors with greater local aggressiveness and presence of cervical metastasis.

## INTRODUCTION

Larynx cancer is the second most common malign neoplasm in the cervicofacial segment. It occurs in males in the proportion of 3.5:1 in relation to females [1]. Larynx neoplasms present greater incidence from the sixth and seventh decades of life [1, 3]. The presence of lymph node metastasis determines the treatment and prognosis for larynx squamous cell carcinoma (SCC) patients, reducing expected survival by up to 50% [4, 5].

Cadherins are a group of cellular adhesion molecules [6, 7]. Epithelial cadherins (E-cadherins) are expressed in all human epithelial tissues and are concentrated in sites of cell-cell epithelial contact [8], whereas catenins of β and γ type make a direct link with the the cytoplasmic portion of E-cadherin, forming the E-cadherin-catenin complex.

The decrease in expression of the E-cadherin molecule leads to the loss of cellular adherence, cytoplasmatic accumulation of β-catenin and its translocation to the nucleus, stimulating cellular proliferation and invasion of epithelial cells [9], increasing the risk towards adjacent or lymph node dissemination [10–12].

The E-cadherin human gene is located in the chromosome 16q22 [13], a region subject to chromosomal translocation in head and neck squamous cell carcinomas [14].

The immunohistochemical expression of E-cadherin and β-catenin in the primary tumor seems to be useful in the identification of patients with clinically negative neck that are considered at risk for hidden metastasis and would need additional treatment.

Some studies have failed to demonstrate a significant statistic relationship between the reduction of the expression of E-cadherin and tumor clinical-histological characteristics, therefore, new investigations are necessary to confirm if the E-cadherin/caterin complex would be important in the clinical decision.

This study aims to assess the influence of the expression of E-cadherin and β-catenin in patients with early or advanced laryngeal tumor and with the presence or absence of cervical metastasis.

## MATERIALS AND METHODS

The casuistry was made by the analysis of consecutive patients with laryngeal cancer in a retrospective cohort between 1996 and 2011.

Inclusion criteria were: patients with glottic or supraglottic squamous cell carcinoma; treated at the Service of Head and Neck Surgery of Hospital Ana Costa de Santos and the Service of Head and Neck Surgery of Irmandade Santa Casa da Misericórdia de Santos; submitted to partial or total laryngectomy with or without neck dissection; therapeutic procedures performed by the same team of surgeons following the same techniques and with curative intent.

Exclusion criteria were: patients whose treatment was radiotherapy, associated or not with chemotherapy; patients submitted to previous surgery and/or oncologic procedures in the superior aerodigestive tract; patients with low histological representativeness in their slides and blocks.

Patients were staged according to the TNM system, eighth edition of the Union for International Cancer Control (UICC, 2017) [15], and assessed according to their age (measured in years), gender (male or female), tumor site (glottic or supraglottic), T stage (T1 to T4), N stage (N0 and N+) and degree of histological differentiation (well differentiated, moderately differentiated or poorly differentiated). Data such as disease-free survival and global survival were also assessed and quantified in months. The data cited were compared with the immunohistochemical expression of the markers E-cadherin and β-catenin.

Slides were obtained by using paraffin blocks of patients treated for the diseases mentioned and stained with hematoxylin and eosin, aiming at proving tumor representation in the material to be analyzed. A tissue microarray (*Beecher Instruments*, Silver Spring, MD) paraffin receptor block was built from original samples (donor block), the area of lesion representation was chosen and marked with a circle, followed by collection with a 1.0 mm diameter needle (*TMArrayer punch* MP10–1.0 mm). A 3 µm thick serial cuts were fixed on to glass slides. Sample staining was performed with hematoxylin and eosin.

Immunohistochemical analysis comprised the evaluation of the expression of polyclonal E-cadherin (clone 36, Ventana Medical Systems Inc.^®^) and β-catenin (Clone 14, Cell Marque^®^). This analysis was performed by using the samples obtained from tissue microarray receptor blocks, which were deparaffinized and prepared by successive immmersion in xylol and ethanol, and submitted to antigen retrieval by heat provided by a pressure cooker, using citrate buffer 10 mM pH6.0. The slides were covered by a saline solution at 4% (3-aminopropyl-triethoxi-silane, Sigma^®^, Saint Louis, USA) diluted in acetone, using the streptovidin-biotin complex (StreptoABC, Dako^®^) to obtain reactions.

With the samples obtained, the endogenous peroxidase was blocked with a solution of hydrogen peroxide at 3% in methanol, immediately after cooling at room temperature for 20 minutes; the slides were rinsed in distilled water. After the peroxidase blocking, immersion took place in Phosphate Buffered Saline solution (PBS).

The slides were incubated with primary antibodies. Subsequently, they were incubated with secondary antibody at titration of 1:200. The use of Diaminobenzidine (DAB, Sigma^®^) solution, sensitive to light and counter stained with Harris (Merck^®^) hematoxylin, showed the reactions. These reactions were accompanied by identification of positive controls, in the absence of primary antibodies, and negative ones when there were no secondary antibodies.

The immunoexpression of every marker was calculated by the counting of one thousand cells, including tumor tissue and non-neoplastic tissue, in every core of the confectioned TMA block and then calculated the percentage of expression to each marker.

Distribution of frequencies was used to describe the category variables (number of cases and percentages) and the measures for central tendency (mean and median) and numeric variables (minimum, maximum and standard deviation). The association between the category variables and the expression measures for E-cadherin and β-catenin was checked through the non-parametric *U* Mann–Whitney test and the Kruskal–Wallis test when the category variable presented 3 categories. The Shapiro-Wilk test was applied to verify the normality of data. Global survival probability and **r**ecurrence free survival probability were estimated by the Kaplan-Meier curve, and the log-rank test was applied to check differences between the survival curves from each variable. A 5% level of significance was adopted for all the statistical tests and the STATA program for computers version 10.0 was used for the performance of the statistical analyses.

## RESULTS

Three patients were excluded for not presenting histological representation after the staining with hematoxylin and eosin, and 4 others were also excluded after material loss during the TMA production.

Table 1 describes selected patient clinical data and their clinic pathological characteristics as well the separation of study groups by site and local progression, with 15 cases in each group. The average age was 63 years with 73.3% of the male gender, 66% of glottic site, 62.2% locally advanced and 44.4% with the presence of lymph node metastasis.

**Table 1:** Distribution of cases according to demographic and clinical variables (*n* = 45)

Variable	Category/Measures	Freq. (%)/Measures
Age (years)	Variation	47–79
	Median	62
	Average (standard deviation)	63.0 (8.2)
Gender	Male	33 (73.3)
	Female	12 (26.7)
Study groups	Advanced glottic tumor	15 (33.3)
	Advanced supraglottic tumor	15 (33.3)
	Early glottic tumor	15 (33.3)
Tumor site	Glottic	30 (66.6)
	Supraglottic	15 (33.3)
T stage	T1	12 (26.6)
	T2	5 (11.1)
	T3	16 (35.6)
	T4	12 (26.6)
T stage	T1 + T2	17 (37.8)
	T3 + T4	28 (62.2)
T stage + glottic site	Glottic-(T1 + T2) Glottic-(T3 + T4)	15 (50.0) 15 (50.0)
T- stage + supraglottic site	Supraglottic-(T1 + T2) Supraglottic-(T3 + T4)	2 (13.3) 13 (86.7)
*N* stage	N0	25 (55.6)
	N+	20 (44.4)
Degree of histological	Well	19 (42.2)
differentiation	Moderately	11 (24.4)
	Poorly	15 (33.3)
Follow-up time	Variation	1–185
(months)	Median	62
	Average (standard deviation)	54.5 (40.1)
Death	No	25 (55.6)
	Yes	20 (44.4)
Time for the occurrence of	N Variation	20 3–33
loco-regional recurrence	Median	9.0
	Average (standard deviation)	10.2 (6.3)
Loco-regional recurrence	No	25 (55.6)
	Yes	20 (44.4)

The distribution of cases according to the markers analyzed is described in Table 2. The average frequency expression for E-cadherin was 62.1% and 44.3% for β-catenin.

**Table 2:** Distribution of cases according to the expression of E-cadherin and β-catenin (*n* = 45)

Variable	Category/Measures	Freq.%/Measures
E-cadherin	Variation	7.9–100.0
	Median	68.4
	Average Standard deviation	62.1 33.6
E-cadherin expression Median cut off point	≤68 >68	22 (48.9) 23 (51.1)
β-catenin	Variation	0–100
	Median	51.0
	Average Standard deviation	44.3 38.2
β-catenin expression Median cut off point	≤50.0 >50.0	21 (46.7) 24 (53.3)

**Figure 1:**
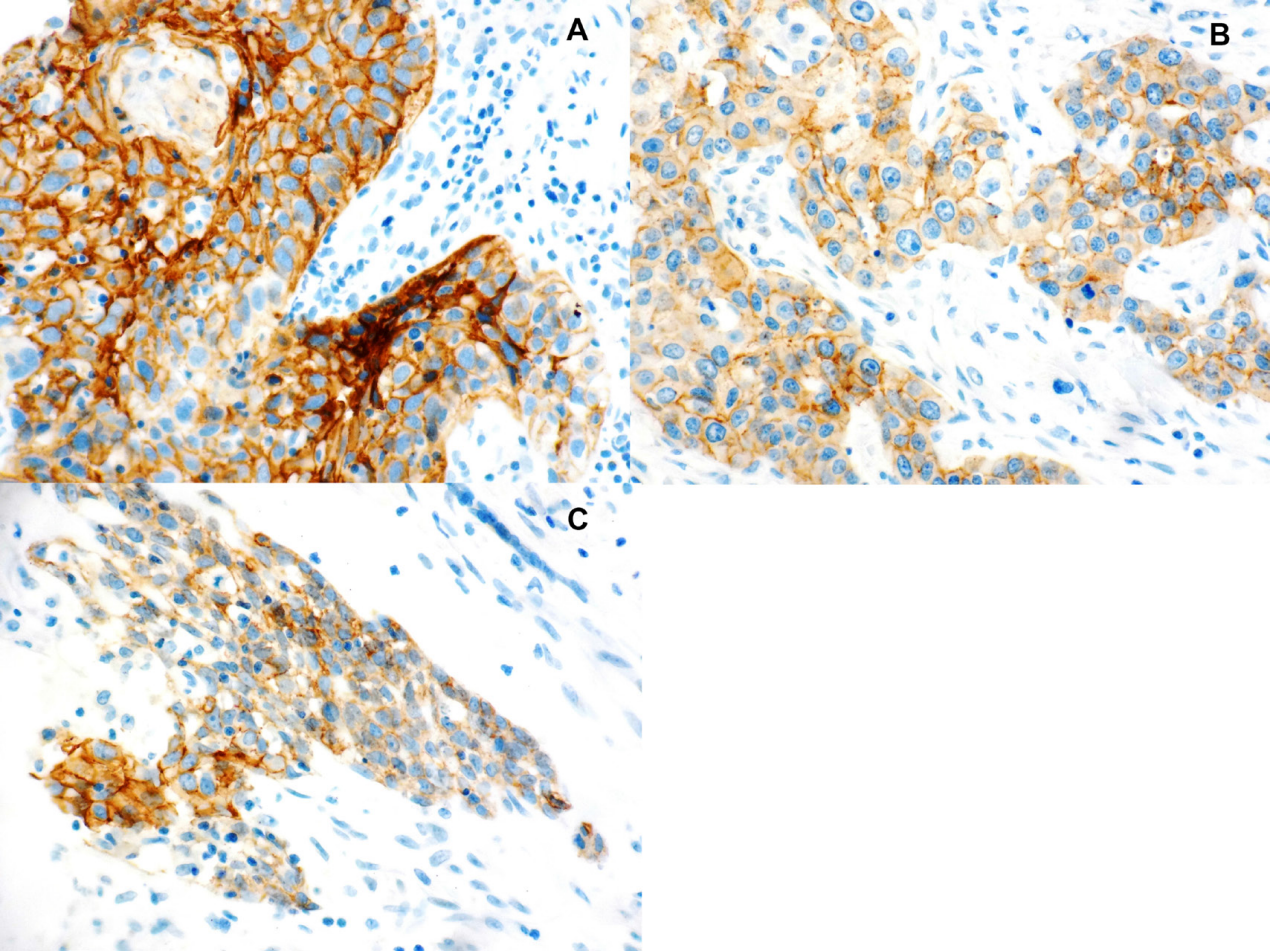
Immunohistochemical staining for E-cadherin expression (**A**) Early glottic squamous cell carcinoma (40×). (**B**) Advanced glottic squamous cell carcinoma with nodal disease (40×). (**C**) poorly differentiated glottic squamous cell carcinoma (40×).

Table 3 shows the associations between the measures of the studied variables according to E-cadherin expression. We observed statistical significance in the case of a reduction in E-cadherin expression in advanced glottic tumors, when compared to early ones, in the occurrence of cervical metastasis and in poorly differentiated tumors (Figure 1).

**Table 3:** Distribution of cases according to E-cadherin expression

Variable	Category	E-cadherin (expression)					*p*-value
		*N*	variation	median	Average	SD	
Age range (years)	≤62 >62	23 22	11.2–100.0 7.9–100.0	81.5 63.4	66.2 57.8	33.8 33.5	0.339
Gender	Female	12	15.3–100.0	52.0	58.9	35.6	0.979
	Male	33	7.9–100.0	72.1	63.3	33.3	
Tumor site	Glottic	15	19.2–100.0	68.4	66.2	21.6	<0.001
(T3 + T4)	Supraglottic	13	7.9–100.0	19.1	25.8	24.5	
T stage + glottic site	Glottic- (T1 + T2) Glottic-(T3 + T4)	15 15	20.0–100.0 19.2–100.0	96.1 68.4	87.1 66.2	23.4 21.6	0.004
T stage + supraglottic site	Supraglottic-(T1 + T2) Supraglottic-(T3 + T4)	2 13	63.0–100.0 7.9–100.0	81.5 19.1	81.5 25.8	26.2 24.5	0.051
*N* stage	N0	25	19.2–100.0	89.2	77.0	26.9	0.001
	N+	20	7.9–100.0	33.9	43.6	32.3	
Degree of	Well	19	20.0–100.0	96.1	87.8	21.2	<0.001^*^
histological	Moderately	11	22.6–100.0	63.0	57.5	26.9	
differentiation	Poorly	15	7.9–88.6	19.2	33.0	25.0	

*p*-value obtained by the *U* Mann–Whitney; ^*^p-value obtained by the Kruskal–Wallis test; SD = standard deviation.

**Figure 2:**
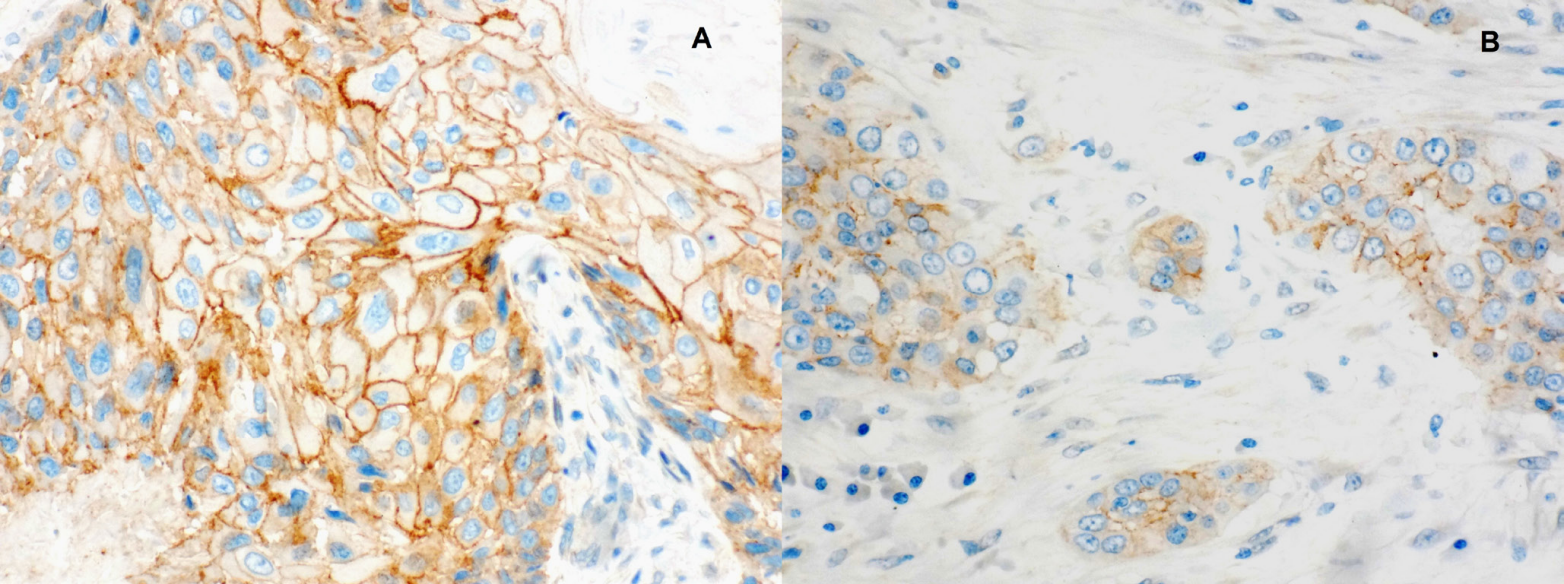
Immunohistochemical staining for β-catenin expression (**A**) Well differentiated glottic squamous cell carcinoma (40×). (**B**) Poorly differentiated glottic squamous cell carcinoma with nodal disease (40×).

Table 4 represents the β-catenin correlation with the selected studied cases. It was observed that low levels of β-catenin are associated with the occurrence of worse tumor differentiation and cervical dissemination (Figure 2).

**Table 4:** Distribution of cases according to the β-catenin

Variable	Category	β-catenin (expression)					*p*-value
		*N*	variation	median	average	DP	
Age range (years)	≤62 >62	23 22	0.0–100.0 0.0–100.0	13.6 56.7	38.3 50.5	40.2 35.9	0.409
Gender	Female	12	0.0–100.0	51.8	47.9	32.8	0.698
	Male	33	0.0–100.0	51.0	43.0	40.4	
Tumor site	Glottic	15	0.0–10.0	67,6	48.7	43.2	0.199
(T3 + T4)	Supraglottic	13	0.0–100.0	11.7	29.0	34.1	
T stage + glottic tumor site	Glottic-(T1 + T2) Glottic-(T3 + T4)	15 15	0.0–100.0 0.0–100.0	56.8 67.6	50.2 48.7	36.8 43.2	0.850
T stage + supraglottic tumor site	Supraglottic-(T1 + T2) Supraglottic-(T3 + T4)	2 13	50.4–81.8 0.0–100.0	66.1 11.7	66.1 29.0	22.2 34.1	0.170
*N* stage	N0	25	0.0–100.0	65.3	56.3	36.3	0.027
	N+	20	0.0–100.0	12.6	29.3	36.0	
Degree of	Well	19	0.0–100.0	60.0	53.1	37.5	0.004^*^
histological	Moderately	11	0.0–100.0	69.7	68.8	31.1	
differentiation	Poorly	15	0.0–79.6	6.9	15.1	24.6	

*p*-value obtained by the Mann–Whitney test; ^*^p-value obtained by the Kruskal–Wallis test; SD = standard deviation.

Data such as global survival in 5 years and recurrence free survival probability in 5 years were also evaluated in relation to E-cadherin and β-caterin markers and are mentioned in Tables 5 and 6, respectively. In this study, we found that lower global survival and lower survival free from recurrence were observed in situations of cervical dissemination and poor histological differentiation, and these were statistically significant. Establishing the median as the cut off point for the expression level, it was found that levels above this point for E-cadherin and β-catenin are associated with better recurrence free survival in 5 years, and for β-catenin with better disease free survival in 5 years.

**Table 5:** Global survival probability in 5 years (*n* = 45)

Variable	*P*	Global survival probability in 5 years (%)	*p*-value
Global survival	(45 patients)	55.3	
Age range (years)	≤62 >62	60.9 50.0	0.652
Gender	Female	75.0	0.111
	Male	48.2	
Tumor site	Glottic	56.7	0.912
	Supraglottic	53.3	
T stage + glottic site	Glottic-(T1 + T2) Glottic-(T3 + T4)	73.3 40.0	0.049
T stage + supraglottic site	Supraglottic (T1 + T2) Supraglottic (T3 + T4)	50.0 53.8	0.925
*N* stage	N0	68.0	0.041
	N+	40.0	
Degree of	Well	68.2	0.007
histological differentiation	Moderately	72.7	
	Poorly	26.7	
E-cadherin expression	≤68 >68	45.4 64.8	0.169
β-catenin expression	≤50 >50	33.3 74.7	0.002

*p*-value obtained by the log-rank test.

**Table 6:** Recurrence free survival probability in 5 years

Variable	Category	Recurrence free survival probability in 5 years (%)	*p*-value
Global Survival	(45 patients)	54.7	
Age range (years)	≤62 >62	56.2 53.0	0.786
Gender	Female	58.3	0.638
	Male	53.5	
Tumor site	Glottic	69.3	0.011
	Supraglottic	26.7	
T + stage + glottic tumor site	Glottic-(T1 + T2) Glottic-(T3 + T4)	92.9 46.7	0.006
T stage + supraglottic site	Supraglottic (T1 + T2)Supraglottic (T3 + T4)	50.0 23.1	0.506
*N* stage	N0	79.2	<0.001
	N+	25.0	
Degree of histological	Well	78.0	<0.001
differentiation	Moderately	72.7	
	Poorly	13.3	
E-cadherin expression	≤68 >68	31.8 77.5	0.002
β-catenin expression	≤50 >50	36.0 70.8	0.005

*p*-value obtained by the log-rank test.

**Figure 3:**
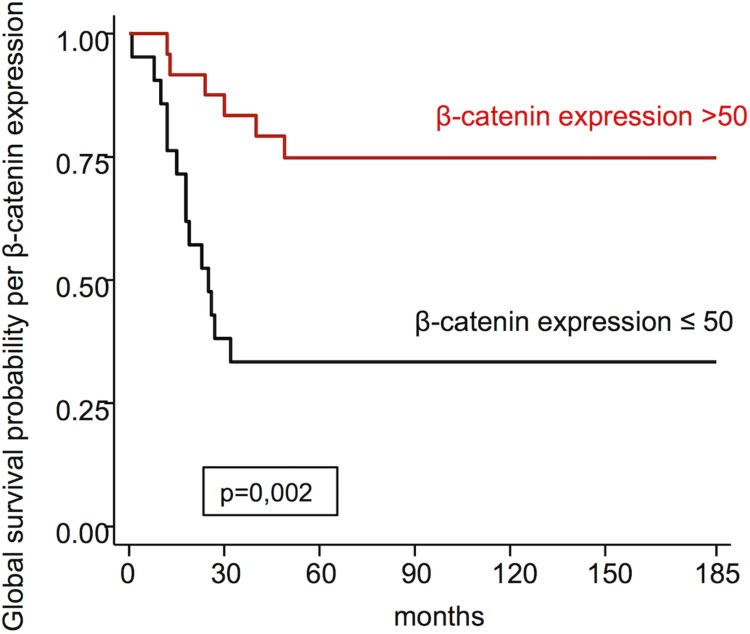
Global survival probability per β-catenin expression

Figure 3 refers to the global survival probability per β-catenin expression using the median as the cut off point. Significance was noticed between the survival curves.

**Figure 4:**
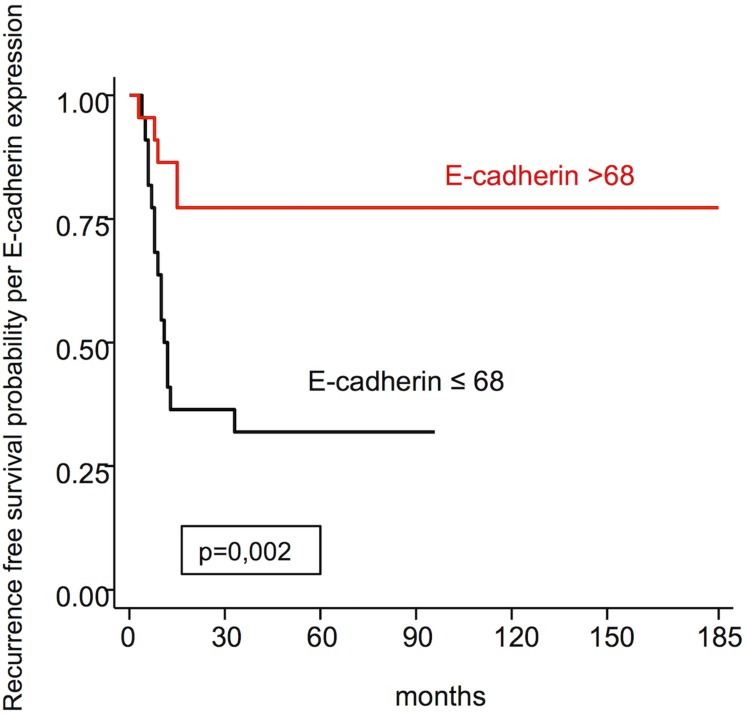
Recurrence free survival probability per E-cadherin expression

**Figure 5:**
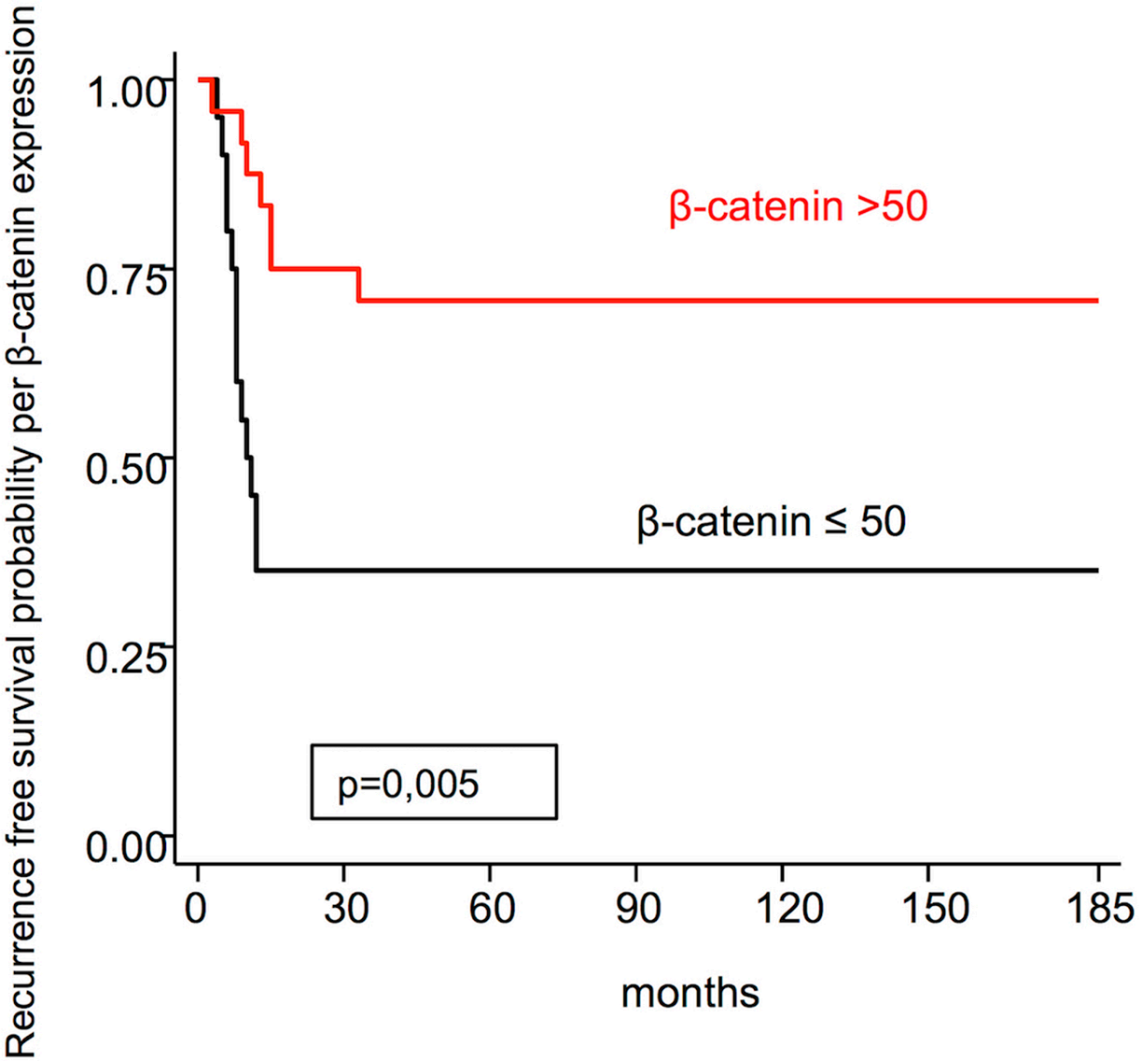
Recurrence free survival probability per β-catenin expression

Figures 4 and 5, respectively, represent the recurrence free survival probability per E-cadherin and β-catenin expression. The difference between the survival curves for both markers show statistical significance.

## DISCUSSION

The marker expression for E-cadherin-catenin complex in laryngeal carcinomas has been studied by immunohistochemical analysis [16]. The presence of this complex is necessary for the maintenance of normal cell adhesion. On this basis, studies proposed that the reduction of this molecule increases the chance of invasion of the cell tissue adjacent to a carcinoma [17, 18]. The reduction in expression of E-cadherin has been correlated with pathological characteristics of the tumor, such as tumor stage, degree of differentiation and lymph node involvement [2].

Studies published between 1996 and 2015 reveal an association between the reduction in E-cadherin expression and cervical metastasis in patients with larynx squamous cell carcinoma [19, 22]. Comparatively, in this study, statistical significance was found in the presence of cervical metastasis in cases of low expression of E-cadherin. While evaluating β-catenin expression, Psyrri *et al.* [23], unlike this study, did not find statistical significance.

Among the studies that evaluated the correlation of the reduction of the above mentioned marker expression with the tumor site, only Ahmed *et al.* [22] and Goulioumios *et al.* [24] found significance when observing less expression of E-cadherin (*p* = 0.002) and β-catenin (*p* = 0.025), respectively, at the supraglottic site. In this present study, it was demonstrated that advanced supraglottic tumors present smaller quantitative expression of E-cadherin when compared to advanced glottic tumors (*p* < 0.001), unlike β-catenin expression, in which there is no such association.

In the evaluation of the tumor measure, revised studies were contradictory, and no statistical correlation with the expression of the markers analyzed was found. In this study, similarly to Psyrri *et al.* [23], not only in glottic, but also in supraglottic cases there was no significance between early and advanced tumors when it comes to variation of β-catenin expression. When the quantitative expression of E-cadherin was evaluated, it was observed that in the glottic site there is significance, with *p* = 0.004. In supraglottic cases, it was noticed that the fall in expression in advanced tumors indicates some association; however, not significant (*p* = 0.051). Ahmed *et al.* [22] and Starska *et al.* [25] found association between the fall in E-cadherin expression and locally advanced tumors.

When the degree of histological differentiation was evaluated, there is statistically significant association, and by the Dunn’s post hoc test, well differentiated tumors present E-cadherin measures higher than in moderately differentiated (*p* = 0.007) and also higher than in the poorly differentiated tumors (*p* < 0.001). Moderately differentiated tumors present higher measures than those poorly differentiated (*p* = 0.031). In the quantitative expression of β-catenin, well differentiated tumors present higher expression than those poorly differentiated (*p* = 0.005), and in cases of moderately differentiated, they were bigger than the poorly differentiated tumors (*p* = 0.001), although there was no relationship with the well differentiated tumors (*p* = 0.184). The presented results were similar to those found in the revised literature, in which there is low differentiation when the expression of markers E-cadherin [19, 21–23, 26, 27] and β-catenin [28] is smaller.

Few studies evaluated the relationship between β-catenin and larynx tumor behavior. Among them, Psyrri *et al.* [23] when assessing 289 patients with malignant laryngeal neoplasm did not find any statistical relationship with tumor progression, lymph node involvement and degree of histological differentiation. Only Lopez-Gonzalez *et al.* [28], with 38 cases, found association between tumors of poorer differentiation with the fall of β-catenin expression.

When assessing global survival and disease free survival, it was found that advanced glottic tumors, the presence of cervical metastases and little differentiation present anegative influence in the prognosis, and the reduction of E-cadherin expression is related to all these events; a similar finding is reported by Li *et al.* [20] and Ahmed *et al.* [22].

The group in which there was absence of metastatic lymph nodes presented greater global survival (68%) and disease free survival (79.2%) in relation to cases with the presence of lymph node metastases (40 and 25%, respectively), this association being of statistical significance (*p* = 0.041 and *p* < 0.001, respectively) and similar to what was found in literature [29].

Greco *et al.* [29] found significant correlation between the increase in E-cadherin expression and the risk for worse global survival. This data was controversial, since the increase of E-cadherin expression would lead to the stabilization of the E-cadherin/β catenin complex. In the present study, we have not found significance in this association.

Using the median as the cut off point, we find that β-catenin expression >50 presents greater global survival (74.7%) than β-catenin ≤50 with survival in 5 years of 33.3% and this difference between the survival curves was statistically significant (*p* = 0.002); this relationship was only evaluated by the study of Greco *et al.* [29], who did not find significance.

Regarding disease free survival, the expression of E-cadherin >68, using the median as the cut off point, presented greater percentage for disease free survival (77.5%) than ≤68 (31.8%), and this difference was statistically significant (*p* = 0.002). Ahmed *et al.* [22], Capelleso *et al.* [30] and Li *et al.* [20] also found a positive relationship between disease free survival and high E-cadherin expression. β-catenin >50 presents greater disease free survival (70.8%) than β-catenin ≤50 (36.0%) and this difference between the survival curves was statistically significant (*p* = 0.005), similar to Greco *et al.* [29].

Considering that the presence of cervical metastasis is an important factor for unfavorable prognosis, cases where there is low expression of the cited markers, which would lead to a greater risk of cervical metastasis, should be assessed regarding a more aggressive treatment and with short-term follow-up, especially those in which there was no indication for previous neck dissection. Another prognostic factor such as tumor progression was also verified where the low E-cadherin expression could represent locally advanced cases in the glottic site. Likewise, cases in which the markers expression is preserved, would tend to have a better differentiation and, therefore, better prognosis.

## CONCLUSIONS

The reduction in expression of the E-cadherin and β-catenin markers in patients with malignant laryngeal neoplasm leads to a prognostic impact when related to the occurrence of cervical metastasis and greater local aggressiveness for E-cadherin in glottic tumors.
